# Miscellaneous

**Published:** 1888-03

**Authors:** 


### MISCELLANEOUS.

### IMPORT DUTIES ON SURGICAL INSTRUMENTS.

At the annual meeting of the Georgia Medical Society, held January 3, 1888, the following resolution was unanimously carried:

*Resolved*, That the Corresponding Secretary enter into correspondence with the medical journals of the country, in order to enlist their influence in support of the movement to remove the import duties from all medical and surgical instruments and appliances, including those used in the diagnosis as well as treatment of disease, so that they may be furnished to those needing them at the lowest possible price.

In compliance with the above resolution, I wish to solicit your earnest attention, and a notice in your publication, which will claim the attention of your readers, hoping that your country readers, especially, will appreciate the truth and importance of our proceedings.

Perhaps the statement of a few facts will assist the reader in realizing the extent of the grievance, and the justice of the plea for which we ask co-operation.

1. Physicians are at the mercy of instrument makers in regard to price, make, and quality of finish, because of the lack of sufficient competition.

2. The price of instruments made in this country is out of proportion to that paid for similar instruments on the Continent of Europe.

3. Surgical instruments and appliances are so costly that but few doctors entering the profession can provide themselves with an outfit adequate to carry on a general practice. At present prices, it is impossible for a country physician's income to sustain his investing in costly instruments, and as a result, many simple cases, such as retention of urine, foreign bodies in nose or throat, deep-seated abscesses, etc., all of which could be relieved at once with the proper instruments, must either die from the immediate cause, or from the effects of time lost in seeking skillful manipulation, or else they are frequently crippled and disfigured, because the most intelligent help, though patiently given, is itself crippled for want of proper instruments.

4. The cheaper grades of instruments are either antiquated, or so poorly made that they may prove a cause of failure in operations, sapping, as it were, the natural inclinations to surgery in its inception.

5. European instruments are from 25 to 75 per cent. cheaper than ours, and their introduction into the market will enable the mass of doctors to buy those of prime necessity, will bring down the price of home-made appliances, and oblige the makers to use good material, and put a better finish to their work.

6. The removal of import duties on surgical and other instruments used by the profession, and on medicines in general, will produce the same results as we all know it did on the article of quinine.

---

### THE WILLIAM F. JENKS MEMORIAL PRIZE.

The first triennial prize, of two hundred and fifty dollars, under the deed of trust of Mrs. William F. Jenks, will be awarded to the author of the best essay on "The Diagnosis and Treatment of Extra-uterine Pregnancy."

The conditions annexed by the founder of this prize are, that the "prize or award must always be for some subject connected with obstetrics, or the diseases of women, or the diseases of children"; and that "the trust see, under this deed for the time being, can, in their discretion, publish the successful essay, or any paper written upon any subject for which they may offer a reward, provided the income in their hands may in their judgment be sufficient for that purpose, and the essay or paper be considered by them worthy of publication. If published, the distribution of said essay shall be entirely under the control of said trustees. In case they do not publish the said essay or paper, it shall be the property of the College of Physicians of Philadelphia."

The prize is open for competition to the whole world, but the essay must be the production of a single person.

The essay, which must be written in the English language, or if in foreign language, accompanied by an English translation, should be sent to the College of Physicians of Philadelphia, Pennsylvania, U. S. A., addressed to Ellwood Wilson, M. D., Chairman of the William F. Jenks Prize Committee, before January 1, 1889.

Each essay must be distinguished by a motto, and accompanied by a sealed envelope bearing the same motto and containing the name and address of the writer. No envelope will be opened except that which accompanies the successful essay.

The committee will return the unsuccessful essays if reclaimed by their respective writers, or their agents, within one year.

The committee reserves the right to make no award if no essay submitted is considered worthy of the prize.

---

LANOLIN.—Since Professor Liebreich extracted oil from sheep's wool and designated it lanolin—wool-oil—it has to a great extent supplanted the petroleum products called vaseline and cosmoline as a basis for ointments in dermatology, because of its ready absorption by the skin. Where a simple lubricating substance is required vaseline and cosmoline answer very well, but where absorption is desired they are less valuable than wool-oil. It is claimed that the objectionable wool-odor which characterized the first preparation of lanolin, can now be obviated without impairing the other qualities of lanolin. Various toilet articles are prepared with lanolin, and designated by such names as *lanolin cold cream*, *lanolin toilet soap*, *lanolin pomade*, etc., etc. As is usual special claims are made for them, by their manufacturers, of possessing exceptional virtues. A substance called *agnine*, which is supposed to be only another name for wool-oil, is also sold.

---

RUSH MEDICAL COLLEGE.—The forty-fifth annual commencement exercises of Rush Medical College, the Medical Department of the Lake Forest University, were held on February 21st. The graduating class numbered 132.

The following is the list of the graduates:

Barnes, Allen C.
Barnes, Edgar Cole
Beeson, Job Strother
Benz, Henry Andrew
Best, Elmer Howard
Bir sall, George Asa
Blim, Charles
Bluthardt, Oscar Robert
Boswell, Davis
Bowlby, Geo. Balfour
Brasington, E. C., M. D.
Brown, Martin Millard
Cantrell, Thomas D.
Carman, Frank W.
Carr, Andrew
Casey, Joseph M.
Cauble, Willis Benton
Cavett, Robert Wm.
Challoner, Robert
Chance, Norman W.
Cherrie, Martin B.
Collins, Wm. P., B. S.
Conaway, John D.
Corley, Charles J.
Dearborn, Henry J.
Defrees, Henry J.
Derham, James E.
Detweiler, Edwin S.
Dolph, Cassius M.
Doolittle, John Comber
Doty, Charles Willard
Dove, Joseph D. F.
Ehle, Hiram Barber
Emerson, Wm. Jesse
Fell, Joshua Harlan
Garber, F. W., B. S.
Gaston, James B.
Getch, Ernst A.
Goddard, James Bell
Goit, Edgar Grant
Goodner, Ralph A.
Grant, Geo. Herbert
Halloran, Florence J.
Hamill, Edwin
Hanna, Harry Howard
Hanson, Frank
Harms, Henry
Heidner, Gustav Adolph
Herrick, J. B., A. B.
Herrmann, Arthur John
Hill, Thomas Caldwell
Hoover, Walker Karl
Hontz, William Cyrus
Howard, Edmund J.
Huberti, Joseph
Hughes, Albert L.
Iles, Urban Grant
Ingalls, Francis Marion
Innis, James Harvey
Irwin, George Howard
Jespersen, Thomas
Jones, Richard R.
Jurgens, Ludwig W.
Kirkpatrick, John W.
Kratochvil, George
Lane, Herbert Warren
Lange, Ignatz
Larson, Carl Frithiof
Lee, Maskel
Loughridge, Victor E.
Lovell, Frank Blair
Marston Ernest L.
Martin, William Brown
Martin, William Giles
Mattox, William R.
Maxwell, George B.
May, James Wallace
McClelland, Clarence B.
McCorkle, George Earl
McGauran, Michael S.
McGrath, John Joseph
Meath, Augustus H.
Moeller, John
Montgomery, Frank H.
Moore, Charles Fred
Munger, Clifton Deo
Murphy, Edward A.
Nelson, Herbert H.
Noble, Wm. L., B. S.
O'Malley, Michael Paul
Owsley, Frederick D.
Perekhan, John Said
Phillips, Carl Fremont
Phillips, George S.
Pitman, Samuel M.
Power, Howard L.
Quirk, John Joseph
Rahlfs, Theodore
Rawlins, John Aaron
Reece, James Nelson
Reynish, D. J., B. S.
Rick, Joseph B.
Ring, John
Richardson, John F.
Saint Cyr, Emilien D.
Sattra, Ole Magneson.
Schoenneshoefer, W.
Schubert, John J.
Schwandt, Emil J.
Seehuus, Ole Martin
Shambaugh, Levi D.
Sherwood, Francis R.
Sims, Luther M.
Smith, J. M., M. D.
Stafford, E. A., A. B.
Steele, Corwin James
Stockwell, John S.
Strickland, C. O., B. S.
Taylor, Fred L.
Taylor, John Dan
Thomas, Charles D.
Titus, William H.
Trask, Howard P.
Vaughn, Phillips C.
Werner, Henry
Wieland, Frank W.
Wilcox, Collin H.
Wiley, Frank A.
Wittman, Adolph R.
Wittwer, Herman R.
Yates, George F.
Yohe, Alfred F.

---

PROPOSED LARYNGOLOGICAL SOCIETY.—A new society is in course of organization in London, as a sequel to the resolution passed by the Subsection of Laryngology and Rhinology at the Dublin meeting of the British Medical Association. The Chairman, Dr. W. McNeill Whistler, in his opening address on that occasion, dwelt strongly on the advantages that such a society would afford to workers in these special branches, who at present have no means of bringing their results to the test of direct criticism by competent judges, except at the annual meetings of the association. It was decided that steps should be taken to carry Dr. Whistler's suggestion into effect, and Dr. R. A. Hayes, of Dublin, was intrusted with the duty of making the necessary arrangements. The list of original members already comprises about fifty names, which include those of nearly all of the prominent laryngologists in the three kingdoms. The following gentlemen have signified their intention of joining the society: Sir Morell Mackenzie, Dr. Whipham, Dr. E. Woakes, Dr. Prosser James, Dr. A. Orwin, Dr. Coleman Jewell, Dr. Greville Macdonald, Dr. Dundas Grant, and Messrs. Lennox Browne, Carmalt Jones, George Stoker, W. R. H. Stewart, and Percy Jakins, of London; Mr. C. Warden, of Birmingham; Dr. Ward Cousins, of Portsmouth; Mr. Creswell Baber, of Brighton; Dr. P. McBride and Dr. G. Hunter Mackenzie, of Edinburgh; Dr. T. Barr, of Glasgow; Dr. Philip Smyly, and Messrs. Kendal Franks, Thornley Stoker, and J. B. Storey, of Dublin; Dr. Walton Browne, of Belfast; and Dr. A. Sandford, of Cork.

---

UNMERITED SYPHILIS.—M. Fournier, in a recent communication, has set forth the statistics which he has taken the trouble to collect of "unmerited" cases of syphilis. In 842 out of 887 infected women, the disease was of venereal origin, leaving 45 who had contracted it in some other way. On analyzing the latter group he found that in seven the disease was hereditary; four had contracted it accidentally in infancy; eight were wet-nurses who had been infected by syphilitic infants; five were midwives who had caught it in the practice of their profession; twenty-two were cases of "domestic infection," either from nurse to child or *vice versa*, or from diseased servants; two of vaccinal syphilis; two in which the infection was conveyed in catheterizing the Eustachian tube; one consequent on rape; and finally four of unknown origin, but certainly independent of sexual contamination. With respect to the first group of 842 infected women, 366 were "gay" women; 220 were married women, and 256 were of "doubtful" social status. Of the married women no fewer than 164 had taken the disease from their husbands. In view of these figures, M. Fournier maintains that the doctrine which forbids discrimination between the different groups of sufferers is one to be unhesitatingly condemned.—*British Medical Journal.*

---

THE DISCIPLINARY POWERS OF THE ROYAL COLLEGE OF PHYSICIANS.—It is stated that at the next meeting of the Comitia of the Royal College of Physicians of London, a resolution will be moved by the Senior Censor requiring that no Fellow, Member, or Licentiate should contribute articles on professional subjects to journals professing to supply medical knowledge to the general public, or should in any way advertise himself or permit himself to be advertised in such journals.

---

THE AMERICAN ANTHROPOLOGIST, a new quarterly journal, published under the auspices of the ANTHROPOLOGICAL SOCIETY OF WASHINGTON, has just been issued. It is edited by a committee consisting of seven members. If the initial number be a fair index of what its future is to be, it will be a creditable addition to the periodical literature of our country.

